# Mistranslation Drives Alterations in Protein Levels and the Effects of a Synonymous Variant at the Fibroblast Growth Factor 21 Locus

**DOI:** 10.1002/advs.202004168

**Published:** 2021-05-01

**Authors:** Ali Bayoumi, Asmaa Elsayed, Shuanglin Han, Salvatore Petta, Leon A. Adams, Rocio Aller, Anis Khan, Carmelo García‐Monzón, María Teresa Arias‐Loste, Luca Miele, Olivier Latchoumanin, Shafi Alenizi, Rocio Gallego‐Durán, Janett Fischer, Thomas Berg, Antonio Craxì, Mayada Metwally, Liang Qiao, Christopher Liddle, Hannele Yki‐Järvinen, Elisabetta Bugianesi, Manuel Romero‐Gomez, Jacob George, Mohammed Eslam

**Affiliations:** ^1^ Storr Liver Centre Westmead Institute for Medical Research Westmead Hospital and University of Sydney Westmead NSW 2145 Australia; ^2^ Section of Gastroenterology and Hepatology PROMISE University of Palermo Palermo 90133 Italy; ^3^ Medical School Sir Charles Gairdner Hospital Unit University of Western Australia Nedlands WA 6009 Australia; ^4^ Gastroenterology Hospital Clinico Universitario de Valladolid School of Medicine Valladolid University Valladolid 47002 Spain; ^5^ Liver Research Unit Instituto de Investigacion Sanitaria Princesa University Hospital Santa Cristina CIBERehd Madrid 28009 Spain; ^6^ Gastroenterology and Hepatology Department Marqués de Valdecilla University Hospital Santander 39008 Spain; ^7^ Department of Internal Medicine Catholic University of the Sacred Heart Rome 20123 Italy; ^8^ Virgen del Rocío University Hospital Institute of Biomedicine of Seville Sevilla 41013 Spain; ^9^ Division of Hepatology Department of Medicine II Leipzig University Medical Center Leipzig 04103 Germany; ^10^ Department of Medicine University of Helsinki and Helsinki University Hospital and Minerva Foundation Institute for Medical Research Helsinki 00290 Finland; ^11^ Division of Gastroenterology Department of Medical Science University of Turin Turin 10124 Italy

**Keywords:** fibroblast growth factor 21, genetics, metabolic, metabolic associated fatty liver disease

## Abstract

Fibroblast growth factor 21 (FGF21) is a liver‐derived hormone with pleiotropic beneficial effects on metabolism. Paradoxically, FGF21 levels are elevated in metabolic diseases. Interventions that restore metabolic homeostasis reduce FGF21. Whether abnormalities in FGF21 secretion or resistance in peripheral tissues is the initiating factor in altering FGF21 levels and function in humans is unknown. A genetic approach is used to help resolve this paradox. The authors demonstrate that the primary event in dysmetabolic phenotypes is the elevation of FGF21 secretion. The latter is regulated by translational reprogramming in a genotype‐ and context‐dependent manner. To relate the findings to tissues outcomes, the minor (A) allele of rs838133 is shown to be associated with increased hepatic inflammation in patients with metabolic associated fatty liver disease. The results here highlight a dominant role for translation of the FGF21 protein to explain variations in blood levels that is at least partially inherited. These results provide a framework for translational reprogramming of FGF21 to treat metabolic diseases.

## Graphical abstract

Paradoxically, fibroblast growth factor 21 (FGF21) levels—a hormone with beneficial effects on metabolism—are elevated in metabolic diseases. It is shown that the primary event is elevation of FGF21 secretion via mistranslation in a genotype and context dependent manner. These results provide a framework for translational reprogramming of FGF21 to treat metabolic diseases.

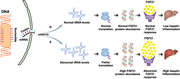

## 1 Introduction

Fibroblast growth factor 21 (FGF21) a liver‐derived hormone that regulates interactions between energy metabolism and stress responses.^[^ ^1^ ^]^ Understanding the actions of FGF21 is complicated by species differences, with most published data coming from animal models; insights on the actions of FGF21 in humans are limited. Paradoxically, despite beneficial effects in rodents, elevated FGF21 levels are reported in metabolic diseases.^[^ ^2^, ^3^, ^4^, ^5^ ^]^ Consistently, FGF21 is reduced in response to corrective interventions such as lifestyle modification and bariatric surgery.^[^ ^1^ ^]^ Whether abnormalities in FGF21 secretion or resistance in peripheral tissues is the initiating factor behind these alterations in humans is unknown. As FGF21 has attracted increasing attention as a target for metabolic diseases,^[^ ^6^ ^]^ clarifying its role in humans is pivotal.

Leveraging human genomic data is an option to resolve the FGF21 paradox and to relate the derived information back to tissue pathology. This is relevant to FGF21 biology for three reasons. First, circulating FGF21 levels are heritable.^[^ ^7^ ^]^ Second, there is large variation in FGF21 levels in healthy subjects, which is highly unusual for bioactive substances^[^ ^8^ ^]^ and suggests that genetic variation in protein synthesis may be important. Third, studies in humans serve as an “experiment‐of‐nature,” overcoming species barriers and the limitation of experimental inaccessibility. Further, genetic studies allow for dissection of the sequence of events that occur at very early time points after the initiation of metabolic perturbations and the development of FGF21 resistance. Finally, interrogation of genetic data allows the study of events in the absence of confounding such as by weight gain and thereby removes (or reduces) the effects of changes in adipose tissue on FGF21 action. This can permit a more direct investigation of the mechanisms of FG21 resistance in humans. Studies through human genetic approaches has been applied to dissect tissue inflammation, and immunity to infection.^[^ ^9^, ^10^ ^]^

Two recent GWAS and subsequent studies have identified pleiotropic and differential effects for variants at the *FGF21* locus, particularly rs838133. This synonymous SNP (sSNP) located in the first exon of the gene is associated with dietary macronutrient and alcohol intake preferences and various metabolic traits including hypertension and total body fat.^[^ ^11^, ^12^, ^13^ ^]^ Studying FGF21 genotype–phenotype associations therefore provides a unique opportunity to study the basis of FGF21 resistance in people.

In this work, we first established that the minor allele at rs838133 mimics the alterations in FGF21 seen in metabolic diseases. We then provide experimental evidence that increased FGF21 production induced by altered mRNA translation kinetics is the likely initial perturbation that increases FGF21 levels in metabolic diseases. We postulate that increased FGF21 induces peripheral FGF21 resistance, while the latter results in a proinflammatory phenotype.

## 2 Results

### 2.1 Increased Serum Fibroblast Growth Factor 21 in Metabolic Associated Fatty Liver Disease Is Correlated with Hepatic Inflammation

To confirm the observation that FGF21 levels are elevated in patients with metabolic associated fatty liver disease (MAFLD),^[^ ^14^, ^15^ ^]^ we assessed its serum levels by enzyme‐linked immunosorbent assay (ELISA) in 410 subjects, including 200 with biopsy‐proven MAFLD and 210 healthy controls. Consistently, an elevation in FGF21 levels was detected in patients with MAFLD compared to healthy controls (Figure S1a, Supporting Information). In addition, circulating FGF21 was elevated in MAFLD patients with diabetes compared to those without (*p* < 0.005) (Figure S1b, Supporting Information). Similarly, there was a correlation between FGF21 and parameters of glycaemic dysregulation–blood glucose, as well as with insulin resistance as measured by HOMA‐IR (*p* < 0.03, for both comparisons, Figure S1c, Supporting Information).

The elevation in FGF21 levels was more profound with advanced MAFLD. An elevation in FGF21 levels was detected in patients with metabolic steatohepatitis compared to those with simple steatosis (*p* < 0.001, Figure S1d, Supporting Information). Similarly, levels were elevated in patients with significant fibrosis (F2–F4) compared to those with no or mild fibrosis (*p* < 0.05, Figure S1e, Supporting Information). In addition, positive and significant correlations were observed between FGF21 and liver enzymes (AST and GGT) as indices of liver injury (*p* < 0.005, for both comparisons, Figure S1f, Supporting Information).

We next analyzed publicly available microarray data of healthy liver for genes correlated with FGF21 expression (Figure S1g, Supporting Information). Several genes demonstrated significant inverse correlations with FGF21. The inflammatory cytokines and chemokines CXCR4, CXCR6, CXCL12, CCL21, IL18, IL1R1, IL7, NF‐κβ signaling, and the cytokines TNFSF4, TNFAIP8L3, C1QTNF1, NFKBIA, PDGFRA and PDGFC all demonstrated significant inverse correlations.

In sum, while high FGF21 levels are associated with hepatic inflammation in MAFLD, the opposite is the case in healthy liver where FGF21 levels negatively correlate with inflammatory markers. Similar beneficial effects have been reported in murine data.^[^ ^16^ ^]^

### 2.2 The rs838133 Minor Allele Affects Fibroblast Growth Factor 21 Protein Levels

**Figure 1:**
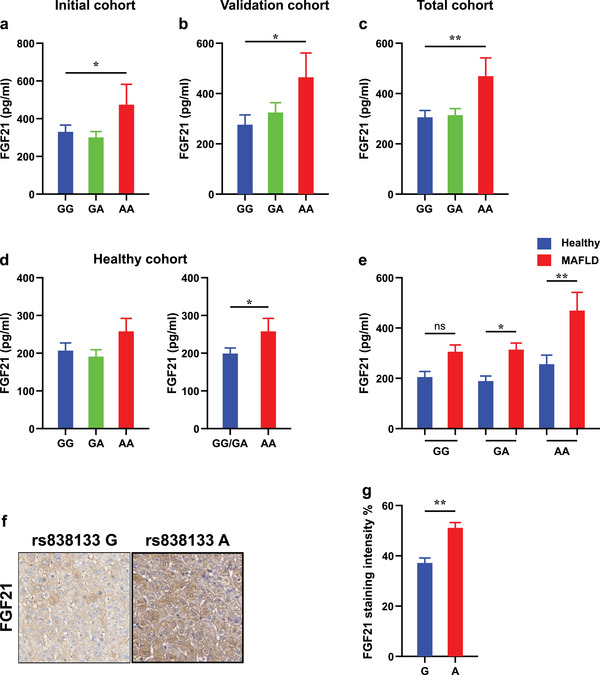
The rs838133 minor allele affects FGF21 protein levels. FGF21 serum levels are elevated in patients carrying the AA genotype compared to those carrying one or two copies of the (G) allele a) in the initial cohort (*n* = 200), b) in the validation cohort (*n* = 211), c) the total cohort (*n* = 411), and d) in the healthy cohort (*n* = 211). e) The changes in serum FGF21 levels between patients with MAFLD (*n* = 411) and healthy controls (*n* = 211) was significant in subjects carrying the (A) allele but not in those with two copies of the (G) allele. Representative liver expression pattern of FGF21 in control subjects carrying the rs838133 AA and GG genotypes. f) Original magnification: 200×. g) The intensity of FGF21 expression in control subjects carrying the rs838133 AA and GG genotypes quantified digitally using ImageJ (*n* = 3, per each group). Statistical differences between groups was assessed by one‐way ANOVA; multiple comparisons were by Bonferroni correction or by *t*‐test, as appropriate (*p < 0.05, ** *p* < 0.01). The data presented are mean ± SEM.

The *FGF21* rs838133 variant has pleiotropic effects on metabolic traits and dietary macronutrient intake preferences; the functional basis of the genetic association is unknown.^[^ ^11^, ^12^, ^13^ ^]^ sSNPs are considered silent or invariant for protein function as by definition they do not alter the amino acid code. However, there is emerging evidence that sSNPs can change protein levels by altering splicing regulatory sites, local mRNA secondary structure, mRNA stability, miRNA binding sites, or translation efficiency.^[^ ^17^, ^18^ ^]^ Having confirmed that FGF21 is increased in MAFLD and with liver inflammation, we assessed the impact of *FGF21* rs838133 on serum FGF21 levels. Unexpectedly, the rs838133 (A) allele was significantly associated with higher serum levels of FGF21 (**Figure** **1**a). This association remained significant after adjusting for age, sex, body mass index (BMI), and diabetes (*β* ± SE: 173.748 ± 63.449; *p* = 0.007). This finding was validated in a second cohort of 211 Italian and Australian patients with MAFLD (Figure 1b) and in a pooled analysis of both cohorts (*n* = 411) (*p* <0.001, Figure 1c). Similar findings were observed in healthy controls (*n* = 211), though it was less profound, with two copies of the (A) allele (AA genotype) required to have a significant effect (Figure 1d). Collectively, the findings are consistent with a previous report showing that the rs838145 G‐allele (in high linkage disequilibrium with the rs838133 A‐allele) (*r* ^2^ = 0.59, *D*′ = 0.78) is associated with higher fasting levels of FGF21 in healthy volunteers from the Baltimore Longitudinal Study of Aging (13).

We next asked whether rs838133 affects the relative change in FGF21 between healthy and metabolic liver disease subjects. Interestingly, the degree of increase in FGF21 protein was significant in patients harboring the (A) allele but not in those with the GG genotype (Figure 1e).

Circulating FGF21 is derived largely from liver;^[^ ^19^ ^]^ to confirm this finding in vivo, we examined FGF21 expression according to rs838133 genotypes by immunohistochemistry. We opted to do this in the liver of control subjects to minimize the confounding impact of concomitant liver disease. Consistently, higher levels of FGF21 protein was found in the liver of people carrying the minor (A) allele compared to those homozygous for the major (G) allele (Figure 1f,g, *p* = 0.0001).

In total, the rs838133 minor (A) allele is associated with higher hepatic FGF21 protein levels in healthy controls and in patients with metabolic disease (MAFLD), though its impact is more profound in the latter.

### 2.3 The rs838133 Minor Allele Increases Hepatic Fibroblast Growth Factor 21 Protein by Increasing Translation

**Figure 2:**
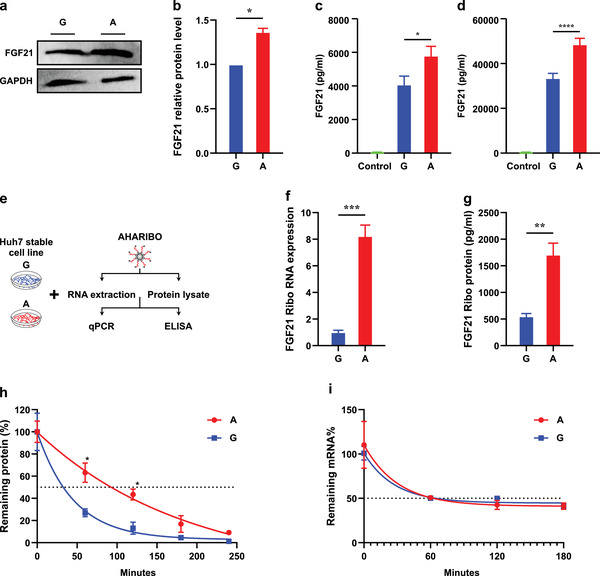
rs838133 minor (A) allele increases FGF21 protein expression via increasing translation efficiency and reducing protein degradation. Huh7 cells stably expressing either the A or G alleles of rs838133 of FGF21 were analyzed. a) Western blot analysis of FGF21 and GAPDH. b) Graph represents quantification of Western blots with fold change compared to the (G) allele. FGF21 protein level measured by ELISA in c) cell lysate and d) secreted FGF21 in medium. e) Illustration of the experimental workflow to study translational efficiency. f) qRT‐PCR of FGF21 mRNA normalized to GAPDH in the ribosomal fraction in the A and G alleles. g) ELISA analysis of FGF21 association with active ribosomes in the A and G alleles. Cells were transiently transfected with the G or A allele during the cycloheximide‐chase assay. h) Results of one‐phase decay after treatment with cycloheximide (CHX, 100 µg mL^−1^) for FGF21 in the A and G allele; half‐life determinations are shown from three independent transfections (A allele, ≈129.7 min, G allele, ≈31.48 min). i)Huh7 cells stably expressing the A and G alleles of rs838133 of FGF21 were analyzed for stability of FGF21 mRNA. Cells were treated with actinomycin D to arrest new transcription and is presented as mRNA remaining over time relative to that at 0 h set as 100%. Half‐life (50% mRNA remaining [dashed line]): A allele, 30.52 min; G allele, 27.46 min. Values of three independent replicates are represented by vertical bars and are mean ± SEM; *p < 0.05 using the student *t*‐test.

Studying FGF21 genotype–phenotype associations can provide an opportunity to study the consequences of allelic alterations in the genome. To address this, we developed Huh7 cell lines that stably express FGF21 isoforms representing the two alleles (A/G) of rs838133. We then examined if the isoforms influence steady‐state FGF21 protein levels. Interestingly, protein expression of the FGF21 minor (A) allele‐expressing cells was increased 25–30% by Western blot (**Figure** **2**a,b, *p* < 0.5) and by ELISA of both cell lysates (Figure 2c, *p* < 0.05) and secreted FGF21 in the medium (Figure 2d, *p* < 0.05) relative to the major (G) allele. These data provide experimental evidence that increased FGF21 production might initiate FGF21 resistance in a genotype‐dependent manner.

We next tried to understand the mechanisms of increased FGF21 production in those harboring the minor allele of rs838133. Four steps contribute to protein abundance, namely transcription, mRNA degradation, translation, and protein degradation. Several reports suggest that protein abundance is predominantly regulated at the level of translation and to a lesser extent by transcription,^[^ ^20^, ^21^ ^]^ while mRNA and protein degradation each only accounts for 5–8% of the variance in protein levels.^[^ ^22^ ^]^ If the rate for one of these steps differs markedly in relation to the others, it would be the dominant mechanism determining variations in protein abundance.^[^ ^23^ ^]^ Hence, we queried these steps to explore the impact of the sSNP rs838133 on FGF21 protein levels.

As demonstrated in Figure S2a, Supporting Information, no difference was observed in the steady‐state mRNA expression level between both alleles of rs838133. Next, we examined if the rs838133 variant alters FGF21 mRNA allele‐specific expression (a method that identifies subtle differences in the effects of variants on gene expression and transcript levels^[^ ^24^ ^]^) in 13 human liver samples from subjects harboring the rs838133 heterozygote genotype. Again, no significant rs838133 allelic differences in FGF21 gene expression was observed (*p* = 0.3, Figure S2b, Supporting Information). This result is consistent with previous observations which report no association between the *FGF21* rs838133 allele and FGF21 expression quantitative trait loci in the liver based on 1031 samples.^[^ ^11^ ^]^

Some sSNPs may alter mRNA splicing by changing the regulatory motifs of exonic splice enhancers.^[^ ^25^ ^]^ To determine if rs838133 affects FGF21 mRNA splicing, we queried the exonic splicing enhancer finder. This revealed that the rs838133 SNP was not predicted to induce any detectable alternative splicing product above the background of the major‐type allele (Figure S2c, Supporting Information). In total, these observations imply that rs838133 does not affect FGF21 mRNA level or splicing.

The observed increase in protein with similar mRNA levels between rs838133 alleles raised the intriguing possibility that this variant regulates the translation rate. To test this, we employed L‐azidohomoalanine (AHA)‐mediated Ribosome isolation (AHARIBO) in the two FGF21 allele cell lines. This novel methodology examines for active translation based on the incorporation of the non‐canonical amino acid AHA followed by sBlock treatment to stabilize the AHA‐labeled peptides on ribosomes. Active ribosomes and their interactome (RNAs or proteins) are purified by magnetic beads targeting the azido groups incorporated in the nascent chains^[^ ^26^ ^]^ (Figure 2e). Subsequently, mRNAs were extracted from both the ribosomal fractions and the total lysates and used for the relative quantification of FGF21 levels using quantitative polymerase chain reaction (qPCR) with the same set of FGF21 probes. Remarkably, the normalized fraction of FGF21 transcripts associated with active translating ribosomes was higher by eightfold in the minor (A) compared to the major (G) allele (Figure 2f).

Then, to estimate if the impact of rs838133 on translation rate would affect FGF21 protein associated with translationally active ribosome levels, we applied the same methodology as above and the protein was isolated. Interestingly, FGF21 protein levels were higher by approximately threefold in the (A) allele compared with that of the (G) allele (Figure 2g).

In addition, we identified NCBI Gene Expression Omnibus (GEO) databases (GEO: GSE32504 and GSE39036) that contain transcriptomic and genotype profiles of 148 liver samples of Caucasian origin. Transcriptomic data was processed via R software including quantile normalization and log2 transformation. Consistently, volcano plot and gene set enrichment analysis (GSEA) between the (A) allele and GG genotype samples showed overrepresentation of gene levels in translation pathways in the (A) allele (Figure S3, Supporting Information), including translation initiation and translational regulation, cotranslation, and ribosomal proteins. In total, these results demonstrate that the minor (A) allele of rs838133 increases FGF21 translation and hence synthesis.

### 2.4 The rs838133 Minor Allele Increases Protein Stability

Protein expression is determined by both production and degradation. In addition to regulating the sensitivity of FGF21 responses, FGF21 activity could also be regulated by alterations in its stability or half‐life. We thus examined whether rs838133 affects FGF21 protein degradation. To this end we assessed the stability of the protein in the presence of the protein synthesis inhibitor, cycloheximide. To minimize the likelihood that different total amounts of protein influence half‐life calculations, cells were transiently transfected with the rs838133 minor (A) and rs838133 (G) allele plasmids. This allowed for total protein abundance to be equal between the two alleles at the beginning of the cycloheximide‐chase experiment (Figure S4a, Supporting Information, by Western blot and Figure S4b, Supporting Information, by ELISA). Consistent with the increased steady‐state expression, the minor (A) allele exhibited a significantly reduced rate of degradation (*t*_1/2_ ≈ 129.7 min) compared with the major (G) allele (*t*_1/2_ ≈ 31.48 min) (Figure 2h).

We also investigated other factors that could contribute to allelic differences in FGF21 protein abundance apart from FGF21 protein degradation. Since mRNA stability may impact protein abundance,^[^ ^27^ ^]^ we measured the half‐life of mRNA of both alleles after treatment of the stably transfected Huh7 cell lines with actinomycin D, a potent transcription inhibitor. No difference between the half‐lives of both alleles was observed (Figure 2i). The results were identical in an analogous experiment using a transient transfection model of both FGF21 alleles (data not shown).

In total, these results indicate that the rs838133 minor allele elicits significant increases in protein stability in human hepatic cells and consequently influences FGF21 homeostasis.

### 2.5 The rs838133 Minor Allele Has Higher Codon Usage

Since rs838133 impacts translation efficiency, protein synthesis, and FGF21 stability, we investigated for the mechanisms by which the translational rate is altered in an rs38133 allele‐specific manner. To do this we investigated for factors that influence translation kinetics. First, we considered relative synonymous codon usage (RSCU) and calculated the RSCU values of glycine encoding codons in the entire human genome and solely within the FGF21 CDS (Table S1, Supporting Information). This analysis revealed that codon usage is biased toward the minor allele (A) which is over‐represented (RSCU > 1.6) in a gene‐specific manner. This indicates that translation elongation of FGF21 is faster in cells with the rs838133 (A) allele.

Given that synonymous variants are implicated in altering mRNA structure and the latter can affect translation efficiency,^[^ ^28^, ^29^ ^]^ we utilized three different algorithms to predict changes in the RNA structure induced by rs838133. These included the RNAfold WebServer (compares the influence of the variant on the secondary structure of the gene),^[^ ^30^ ^]^ RNAsnp (provides a *p*‐value reflecting significance of local structure base‐pairing distance),^[^ ^31^ ^]^ and MutaRNA (provides a collection of measures and visualizations for an intuitive interpretation of the impact of variants on the RNA secondary structure).^[^ ^32^ ^]^ The three algorithms consistently revealed that rs838133 causes significant secondary structure changes and weak secondary structures within mRNAs in the (A) allele that can accelerate translation^[^ ^33^ ^]^ (Figure S5, Supporting Information).

### 2.6 RPLP0 Abundance Determines Effects of rs838133 on the Translation Efficiency

**Figure 3:**
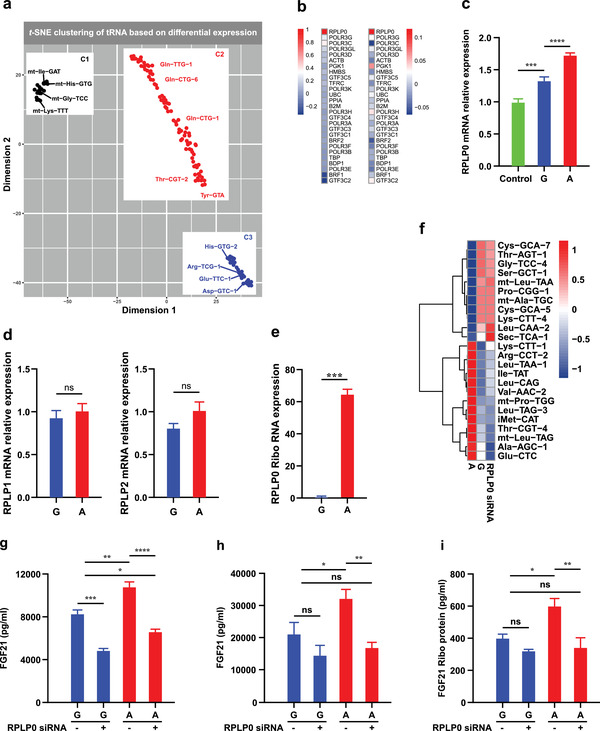
RPLP0 abundance determined effects of rs838133 on translation efficiency. a)Multivariate analysis of the tRNA expression patterns using t‐SNE. Three groups of tRNAs (C1, C2, and C3) are highlighted. b)Heatmap of quantification of change in abundance of transcription of 28 tRNA‐associated genes in Huh7 cells stably expressing the A and G alleles of rs838133 of FGF21 analyzed by TaqMan Array and in GEO: GSE32504 and GSE39036, respectively. This showed a unique upregulation in RPLP0 in A allele‐expressing cells c) that was confirmed by RT‐PCR, d) with no changes in other RPLP proteins (RPLP1 and RPLP2). e) The normalized fraction of RPLP0 transcripts associating with actively translating ribosomes was higher in the minor (A) allele compared to the major (G) allele. f) Hierarchical clustering of tRNAs based on their relative changes in abundance upon RPLP0 inhibition by siRNA. RPLP0 inhibition by siRNA decreases FGF21 protein level measured by ELISA g) in cell lysates and h) secreted in medium and i) in active ribosomes in the (A) allele cells restoring it to levels comparable to that in (G) cells. Values of three independent replicates are represented by vertical bars and are mean ± SEM; *p < 0.05 using the student *t*‐test or one‐way ANOVA; multiple comparisons were by Bonferroni correction, as appropriate.

We investigated other mechanisms by which the translation rate can be altered in an rs838133 allele‐specific manner. Transfer ribonucleic acid (tRNA) is a small ribonucleic acid (RNA) chain (74–93 nucleotides) that transfers a specific amino acid to a growing polypeptide chain at the ribosomal site of protein synthesis during translation. tRNA abundance is a major determinant of ribosome speed at a codon.^[^ ^34^ ^]^ Thus, we measured tRNA levels using nrStar Human tRNA PCR Arrays that profile 185 human tRNAs (163 nuclear tRNA isodecoders and 22 mitochondrial tRNA species) covering all the nuclear anticodon isoacceptors catalogued in GtRNAdb and providing insights on both the isoacceptor and the specific isodecoder expression in the Huh7 cells expressing the two allelic variants. Using t‐distributed stochastic neighborhood embedding (t‐SNE), we found that the tRNAs can be clearly segregated into three clusters (C1, C2, and C3) (**Figure** **3**a). The distribution of tRNAs among the three clusters corresponded to key functional features. C1 is enriched in mitochondrial tRNA while C2 contains increased levels of tRNA for the uncharged group. In contrast, C3 has more charged tRNA with the increase in the tRNA for the uncharged group being less pronounced. These observations indicate that the tRNA pool is rearranged according to the FGF21 allelic variant that influences translation.

To further investigate the basis of this change in the tRNA pool, a human reverse transcription‐quantitative polymerase chain reaction (RT‐qPCR) array was used to screen cells stably expressing the two FGF21 alleles for differential abundance in transcription of 28 tRNA associated genes. Interestingly, this analysis revealed that ribosomal protein lateral stalk subunit P0 (RPLP0) abundance increased substantially in the minor (A) allele‐expressing cells, while other genes levels were unchanged between the two groups samples (Figure 3b). Analysis of GEO: GSE39036 data confirmed that RPLP0 is upregulated in the (A) allele compared to the GG genotype, with no differences in other genes (Figure 3c). The human ribosomal P complex consists of the acidic ribosomal P proteins and includes in addition to RPLP0, both RPLP1, and RPLP2 (RPLP proteins). Therefore, we investigated for these three RPLP proteins in the stable expressing cells of rs838133 alleles using reverse transcriptase‐polymerase chain reaction (RT‐PCR). This confirmed increases in RPLP0 in the minor (A) allele‐expressing cells, while there was no difference in expression of the other RPLP proteins, RPLP1 and RPL2 (Figure 3d), with similar data in the GEO dataset.

Next, we investigated for the differential expression pattern of RPLP0 in the translating ribosomes from stably expressing cells of the (A) and (G) alleles. Remarkably, the normalized fraction of RPLP0 transcripts associated with active translating ribosomes was higher by ≈60‐fold in the (A) compared to the (G) allele (Figure 3e).

Hence, the robust and unique enrichment of RPLP0 levels in the (A) allele caught our interest and we opted to determine whether suppression of RPLP0 by specific siRNA would influence tRNA levels, translation kinetics, and FGF21 levels in human hepatocytes stably expressing the rs838133 minor (A) and rs838133 (G) alleles. In samples transfected with RPLP0‐directed siRNA, RPLP0 mRNA levels were reduced by ≈80%, with no change in other RPLP proteins (RPLP1 and RPL2) (Figure S6a–c, Supporting Information), and no cell toxicity was observed. Interestingly, RPLP0 silencing changed the tRNA profile in cells expressing the (A) allele, restoring it to comparable levels to the (G) allele (Figure 3f). In addition, RPLP0 silencing decreased FGF21 protein in cells expressing the (A) allele, restoring it to comparable levels to the (G) allele in the cell lysate (Figure 3g) and in FGF21 levels in the medium (Figure 3h). However, RPLP0 silencing, only modestly enhanced FGF21 mRNA expression by ≈1.4‐fold. The latter is likely a feedback mechanism suggesting that the effect of RPLP0 on FGF21 is mainly on translation (Figure S6d, Supporting Information). To confirm this, we analyzed whether RPLP0 suppression reduces FGF21 protein associated to translationally active ribosomes levels using the AHARIBO method. Consistently, RPLP0 silencing decreased total FGF21 protein in cells expressing the (A) allele to comparable levels to that of the (G) allele (Figure 3i). We conclude that the RPLP0 depletion effect is primarily through effects on translation and an altered tRNA profile.

### 2.7 The rs838133 Minor Allele Does Not Affect Fibroblast Growth Factor 21 Receptor and Fibroblast Activation Protein Levels

FGF21 acts on a cell surface receptor complex comprised of two proteins: a conventional tyrosine kinase FGF receptor (FGFR) and a coreceptor protein, *β*‐klotho (KLB). FGF21 preferentially binds and activates the FGFR1 that is predominantly expressed in adipose tissue and adipocytes.^[^ ^35^ ^]^ The notion of “FGF21 resistance” is the widely proposed explanation for increases in circulating FGF21 levels in metabolic diseases.^[^ ^36^ ^]^ In this model, the increase in FGF21 is caused by a downregulation of expression of its receptors (or desensitization) mainly in white adipose tissue, resulting in compensatory FGF21 production.

**Figure 4:**
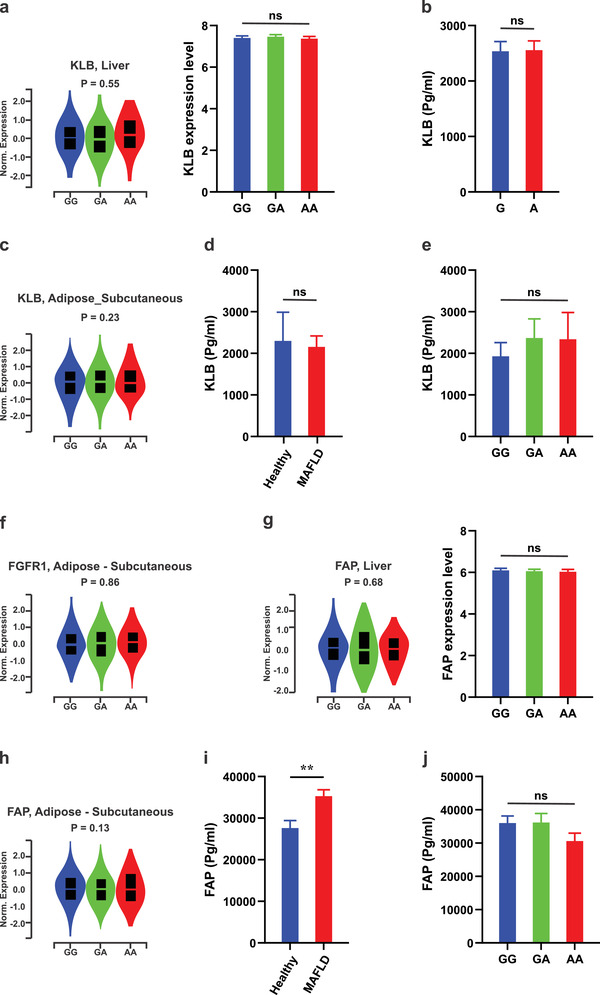
The rs838133 minor allele does not affect FGF21 receptor and FAP levels. a) rs838133 is not associated with KLB gene expression in the liver based on the Genotype‐Tissue Expression (GTEx) data and the GEO dataset (GEO: GSE32504 and GSE39036). b) No difference was observed in steady‐state KLB mRNA expression levels between stably expressing cells of the two alleles of rs838133. c) This variant was not associated with KLB gene expression in adipose tissue from the GTEx database. Circulating KLB concentration was not significantly different between patients with d) MAFLD (*n* = 200) and healthy individuals (*n* = 44), or e) according to rs838133 genotype in patients with MAFLD. f) rs838133 is not associated with FGFR1 gene expression in adipose tissue from the GTEx database. rs838133 is not associated with FAP gene expression in the liver in g) both the GTEx database and the GEO dataset or in h) adipose tissue. i) Circulating FAP concentration was higher in patients with MAFLD (*n* = 200) compared to controls (*n* = 44). j) In patients with MAFLD, FAP serum levels were not associated with rs838133.

Thus, to provide a comprehensive evaluation of the basis of FGF21 abnormalities in metabolic disease, we analyzed the association between rs838133 and the expression of molecular actors of FGF21 responsiveness (FGFRs and KLB) in liver and adipose tissue. There was no evidence that this variant is associated with KLB gene expression in the healthy liver based in the human Genotype‐Tissue Expression (GTEx) database; this was confirmed using the GEO dataset (**Figure** **4**a). Similarly, no difference was observed in steady‐state KLB mRNA expression levels between both stably expressing cells of the two alleles of rs838133 (Figure 4b). In addition, this variant was not associated with KLB expression in adipose tissue in the GETEX database (Figure 4c). For further confirmation, we evaluated circulating KLB in 200 patients with MAFLD and in 44 healthy individuals. Circulating KLB concentration was not different between patients with MAFLD and healthy controls (*p* > 0.5; Figure 4d). Consistently, in patients with MAFLD, KLB levels were not different according to rs838133 genotype (*p* > 0.3; Figure 4e). Similarly, this variant was not associated with FGFR1 gene expression in adipose tissue (Figure 4f).

Human FGF21 is inactivated by fibroblast activation protein (FAP),^[^ ^35^ ^]^ so we also examined the association between rs838133 and FAP levels. As depicted in Figure 4g, this variant was not found to be associated with FAP gene expression in the liver in both the GTEx database and the GEO dataset, or in adipose tissue in the GTEx database (Figure 4h). To verify this finding, we measured circulating FAP in 200 patients with MAFLD and 44 healthy individuals. Circulating FAP concentration was higher in patients with MAFLD compared to controls (*p* < 0.001; Figure 4i). In patients with MAFLD, FAP serum levels were not associated with rs838133 (*p* > 0.8; Figure 4j).

Finally, to investigate if the receptors are equally activated with FGF21 that issues from either the G or A allele, we assessed for FGF21 signaling in both A and G stably expressing cell lines by measuring the FGF21 downstream signaling proteins pERK and pAKT and the expression of Egr1 and cFos, as recommended as the criteria for defining the term “FGF21 resistance."^[^ ^37^ ^]^ As shown in Figure S7, Supporting Information, we did not observe any differences between A and G stably expressing cell lines. In total, this data suggests that despite genotype dependent increases in FGF21 protein production, there is no alteration in FGF21 responsiveness (FGFRs and KLB) in liver or adipose tissue or in FGF21 activity.

### 2.8 The rs838133 Minor Allele Increases the Risk of Hepatic Inflammation in Metabolic Associated Fatty Liver Disease

**Figure 5:**
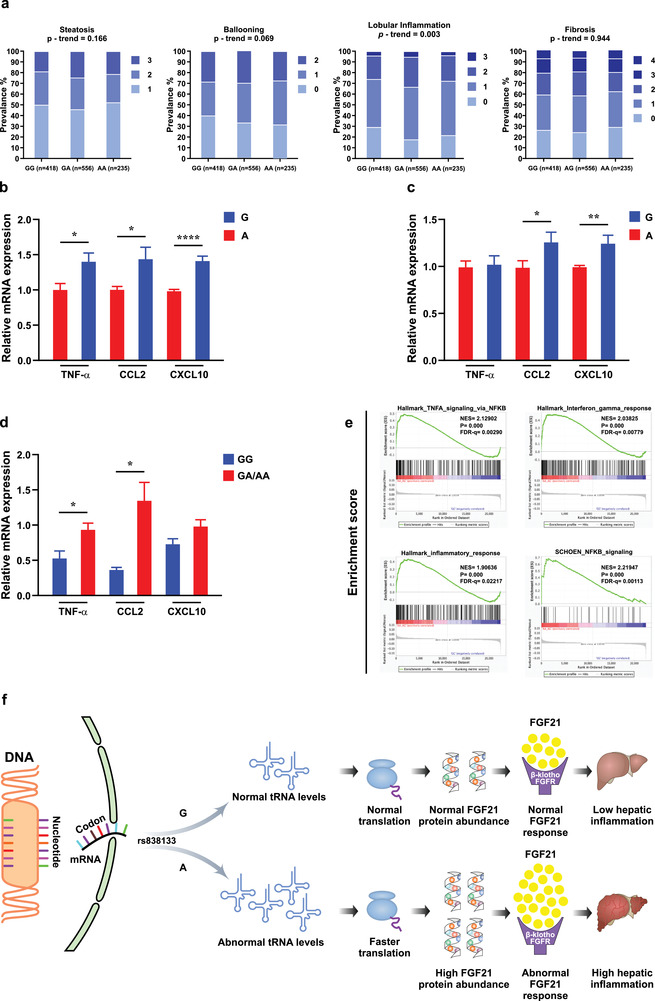
The rs838133 minor allele impacts the acquisition of a proinflammatory phenotype in hepatocytes. a) Histological features were evaluated in a large cohort of biopsy‐proven patients with MAFLD (*n* = 1209). GG, homozygotes for the G allele; GA, heterozygotes; AA, homozygotes for the A allele. Numbers per each genotype are presented under the figure. Genetic analyses were calculated by using an additive model; *p*‐values represent the significance for trend in the prevalence of more severe degrees of histologic damage among the genotypes. Huh7 cells stably expressing the A allele had lower levels of TNF‐*α*, CCL2, and CXCL10 in response to b) TLR4 (LPS, 500 ng) or c) palmitic acid and oleate (200 and 400 um, respectively) after 24 h challenge, compared to the G allele (*n* = 3). d) Comparison between hepatic expression of inflammatory markers between subjects carrying the (A) allele (*n* = 7) or (GG) homozygous genotype (*n* = 3) shows that the minor allele (A) carriers express higher proinflammatory markers (TNF‐*α*, CCL2, and CXCL10). e) Gene set enrichment analysis (GSEA) analysis for the NCBI Gene Expression Omnibus (GEO) database (GEO: GSE32504 and GSE39036) that contains transcriptomic and genotype profiles, respectively, of 149 liver samples of Caucasian origin subjects shows over‐representation of genes encoding inflammatory pathway in the (A) allele versus those with two copies of the (G) allele. f) Proposed model for the functional effects for the synonymous single nucleotide polymorphism rs838133 via mistranslation and alteration of protein abundance. mRNA expression was analyzed by real‐time PCR and normalized to GAPDH. Data are represented by vertical bars and are mean ± SEM; *p < 0.05 using the student *t*‐test. Abbreviations: TNF*α*, tumor necrosis factor alpha; C–X–C motif chemokine 10 (CXCL10) also known as interferon *γ*‐induced protein 10 kDa; Chemokine CCL2 (also known as monocyte chemoattractant protein‐1, MCP‐1).

To relate our findings back to tissues outcomes, we assessed the association of rs838133 with hepatic inflammation. Though *FGF21* rs838133 shows a pleiotropic effect on various metabolic traits,^[^ ^11^, ^12^, ^13^ ^]^ the role of this variant in MAFLD is unknown. Shared risk variants between metabolic diseases are increasingly recognized.^[^ ^38^, ^39^ ^]^ Thus, we genotyped the *FGF21* rs838133 variant in a cohort of individuals (*n* = 1209) with histologically proven MAFLD. Baseline characteristics of the cohort are summarized in Table S2, Supporting Information. *FGF21* rs838133 was confirmed to be in Hardy–Weinberg equilibrium and showed a minor allele frequency of 0.43 in our cohort, similar to that observed in other European populations, (http://browser.1000genomes.org). The association of rs838133 genotype with the spectrum of liver damage related to MAFLD is shown in **Figure** **5**a.

In this analysis, the minor (A) allele was associated with severe hepatic inflammation (*p* = 0.003) using an additive model. The effect was more profound when the cohort was dichotomized into absent (0) versus any inflammation (A1–A3) (*p* = 0.0001) or when adopting a dominant model that compared the presence of any inflammation (Odds ratio [OR]: 1.77; 95% confidence interval [CI]: 1.34–2.33, *p* = 0.0001). The association with inflammation remained significant after adjusting for age, gender, BMI, and type 2 diabetes mellitus (OR: 1.67, 95% CI: 1.29–2.16, *p* = 0.0001). The (A) allele was also associated with severe inflammation (A2–A3) (OR: 1.32; 95% CI: 1.01–1.71, *p* = 0.04). A similar trend was observed with the severity of ballooning though this was not significant (*p* = 0.069). No association was observed with either steatosis or fibrosis. No differences in clinical, anthropometric, or biochemical indices including aminotransferases were found between the *FGF21* rs838133 genotypes.

### 2.9 The rs838133 Minor Allele Has Differential Effects on Inflammation in Hepatocytes In Vitro and In Vivo

Based on the above data, we explored the functional impact of rs838133 on inflammation using Huh7 cells that stably express the two *FGF21* alleles. In this analysis, the rs838133 (A) allele‐expressing cells had lower induction of mRNA levels of key cytokines and chemokines including monocyte chemoattractant protein‐1 (MCP‐1/CCL2), chemokine (C‐X‐C motif) ligand 10 (CXCL10), and tumor necrosis factor alpha (TNF‐*α*) in response to stimulation of the toll‐like receptor 4 (TLR4) by lipopolysaccharide (LPS) (Figure 5b) or palmitate and oleate (Figure 5c) (*p* < 0.05). These findings are in line with the known anti‐inflammatory effects of FGF21.

Our in vivo results were opposite when comparing the liver expression of inflammatory markers in MAFLD patients carrying either the rs838133 (A) or (G) genotype allele. Consistent with the earlier genetic findings, we found enhanced expression of these markers in the liver of patients harboring the (A) allele compared to those carrying the (G) allele (*p* < 0.05, *n* = 11) (Figure 5d). Consistently, GSEA between (A) allele and GG genotype samples in the GEO database shows overrepresentation of gene levels in inflammation pathways in the (A) allele (Figure 5e), including inflammatory response, TNF‐*α* signaling, NFK*β* signaling, and interferon gamma responses. Finally, we investigated the role of rs838133 on the regulation of hepatocyte lipid droplet content. As shown in Figure S6a,b, Supporting Information, no difference in intracellular triglyceride content was noticed with palmitate and oleate treatment in cell lines overexpressing either the *FGF21* A or G alleles.

## 3 Discussion

The nutritional regulation of FGF21 is complex and liver FGF21 production appears to be stimulated by an imbalance in macronutrient intake. In particular, circulating FGF21 levels are markedly increased by diets that are high in carbohydrate but low in protein, as well as by alcohol consumption.^[^ ^40^, ^41^, ^42^ ^]^ In contrast, exogenous FGF21 treatment reduces the consumption of sweet foods and alcohol, and increases the consumption of protein.^[^ ^41^ ^]^ In people, several large genetic studies have shown that the rs838133 minor (A) allele is associated with higher sugar and alcohol intake and lower protein intake.^[^ ^11^ ^]^ Our findings suggest that the metabolic stress induced by a high sugar and low protein western type diet in the (A) allele carriers might trigger the high FGF21 levels we observed. Despite genotype dependent increases in FGF21 protein production, there is no alteration in FGF21 responsiveness (FGFRs and KLB) in liver or adipose tissue or in FGF21 activity. This implies that an early and perhaps primary event in FGF21 alterations with the onset of human metabolic diseases is increasing genotype‐dependent FGF21 production rather than decreased peripheral tissue sensitivity. This might be mediated by RPLP0‐tRNA‐dependent remodeling of the stress‐induced translatome as an adaptive response to suppress the ingestion of simple sugars, while stimulating protein intake. However, further increases in FGF21 with progressive metabolic morbidity could then lead to an imbalance between FGF21 and its occupied receptors leading to impairment of FGF21 downstream signaling (Figure 5f). Whether this effect is tissue specific or a generalized effect is yet to be determined.

In line with our hypothesis, a recent study employed a tissue‐specific *β*‐klotho transgenic mouse and showed that FGF21 resistance is not mediated by downregulation of *β*‐klotho expression in white adipose tissue.^[^ ^43^ ^]^ Similarly, another report demonstrated lack of obvious FGF21 resistance in two mouse models of obesity and insulin resistance, viz ob/ob and diet‐induced obese mice.^[^ ^44^ ^]^ Furthermore, a time‐course study in the heart over 12 weeks after high fat diet feeding demonstrated that increase in plasma FGF21 levels precedes impairment of cardiac FGF21 signaling pathways, including FGFR1, *β*‐Klotho, p‐ERK1/2, p‐Akt, and PGC‐1*α* expression.^[^ ^45^ ^]^

It is noteworthy that similarly for insulin resistance, its signaling pathway has been investigated in detail and two points of view are reported. The classic dogma is that lack of sensitivity in peripheral tissue raises blood‐glucose levels, which in turn stimulates insulin hypersecretion. However, on the basis of numerous observations challenging this traditional paradigm, counter arguments have emerged that postulate the reverse. In this scenario, hyperinsulinemia is the initial and primary abnormality in obesity which drives subsequent insulin resistance.^[^ ^46^ ^]^ This notion is supported by a variety of experimental data including that after high fat diet feeding regimens in both murine and human subjects, in which elevated fasting levels of circulating insulin and not glucose, is the initial event.^[^ ^46^ ^]^ In addition, it is commonly recognized that some people with long‐established obesity demonstrate fasting hyperinsulinemia despite showing no signs of excess blood‐glucose concentrations that would theoretically be required to promote insulin secretion.^[^ ^47^, ^48^ ^]^

Cells dynamically adjust their protein translation profile to maintain homeostasis in the context of changing environmental cues. During nutrient and metabolic stress, the eukaryotic translation initiation factor 2a kinase, general control non‐depressible 2 (GCN2) is activated to elicit a homeostatic response, called the integrated stress response (ISR).^[^ ^49^ ^]^ Acute/transient stress is characterized by decreased global protein synthesis and increased upstream open reading frame (uORF) mRNA translation. Following resolution of the ISR, normalization of protein synthesis occurs.^[^ ^49^ ^]^ However, during chronic metabolic stress, the chronic ISR program is characterized by persistently elevated uORF mRNA translation, with the vast majority (98%) of mRNAs which were translationally upregulated during the acute ISR remaining translationally active.^[^ ^50^ ^]^ ISR activation has been observed in metabolic diseases including MAFLD.^[^ ^51^ ^]^ In the liver, it has been shown that ISR activation in response to diverse physiological or pathological stressors such as starvation, results in a dramatic induction of FGF21.^[^ ^52^, ^53^, ^54^ ^]^ In this context, a direct and pivotal role for RPLP0 in propagating the signal from ribosomes perturbed by nutrient stress (amino acid starvation) to GCN2 and ISR activation has been recently reported, without affecting the endoplasmic reticulum unfolded protein stress‐induced ISR.^[^ ^55^ ^]^ In addition, previous data indicates that although RPLP0 phosphorylation is not required for overall ribosome activity, it may affect the translation of specific proteins, those mainly involved in metabolic processes.^[^ ^56^ ^]^ In total, our data suggests that RPLP0 could be the link between nutrient stress, t‐RNA profile, translational rate, and increased FGF21 levels in metabolic disorders and is regulated at the gene level. In this scenario, reduced downstream FGF21 signaling and activity would be a secondary adaptive response in dysmetabolic states. Further studies would be required to clarify the detailed mechanisms of RPLP0 upregulation of the *FGF21* A allele and the effects on FGF21 levels.

Linking our findings back to tissues, our data implies a mechanistic connection between presence of the (A) allele, elevated FGF21, and an inflammatory phenotype under metabolic stress. We confirm a role for FGF21 as a component of the milieu contributing to steatohepatitis, with the inflammatory phenotype likely representing FGF21 resistance. Interestingly, we demonstrated a differential response for the rs838133 (A) allele in vitro and under metabolic stress in vivo, further supporting our proposed model.

Due to the pleiotropic and multi‐organ metabolic actions of FGF21 and emerging evidence that this hormone mediates the therapeutic effects of currently available drugs and those under development for treatment of metabolic diseases, FGF21 has attracted great interest as a therapeutic target for disorders such as obesity, type 2 diabetes, MAFLD, atherosclerosis, and cardiovascular diseases.^[^ ^2^, ^3^, ^4^, ^5^ ^]^ Intriguingly, our data suggests that approaches to mildly reduce FGF21 production could be a novel and more efficient strategy to treat obesity and metabolic disorders. This could be achieved via targeting its translational reprogramming to allow precise tuning of protein synthesis rates. The levels of circulating FGF21 necessary to mediate beneficial metabolic effects without bone toxicity^[^ ^57^ ^]^ is around 25–50 ng mL^−1^, independent of the target organ. This is much lower than the peak concentrations achieved in clinical trials after periodic administration of FGF21 analogues at supraphysiological doses.^[^ ^58^, ^59^ ^]^ Consistently, some recent studies show that modulation of the translation velocity could be a better therapeutic strategy for the treatment of cystic fibrosis mutants and for some types of cancers.^[^ ^60^, ^61^ ^]^ Further studies would be required to determine the feasibility and safety of this therapeutic approach.

Finally, our data argues for the importance of the incorporation of an individual's genome composition information in clinical trial ecosystems and drug development. This would allow for improved characterization of the target population that would benefit from FGF21 treatment and ensures that the intervention is equally effective across different populations. Such an approach requires moving from traditional one‐phase, one‐drug, one‐indication clinical trials to novel trial platforms that use master or basket protocols.^[^ ^62^ ^]^

In conclusion, we identified an unanticipated mechanism that helps to reconcile the apparently contradictory reports regarding the significance of elevations in FGF21 in the context of metabolic diseases. Our results suggest that the primary event in dysmetabolic states is an initial elevation of FGF21 secretion by translational reprogramming in a genotype and context specific manner. The upregulated FGF21 then induces FGF21 resistance, for example, in white adipose tissue, as a beneficial rather than a pathological adaptation to inhibit FGF21 signaling. An important next step will be to determine whether elevations in FGF21 are coupled to specific changes in FGF21 receptors or to factors binding to the receptors that render it less efficacious following repeated stimulation, and how it can be best targeted to treat metabolic diseases.

## 4 Experimental Section

##### Plasmids

Transfection was performed with FuGENE HD (Promega) according to the manufacturer's instructions. pcDNA3.1 constructs containing the full‐length human *FGF21* with genetic variation corresponding to A or G (of rs838133) were used for in vitro transcription and translation experiments as described previously.^[^ ^28^, ^29^ ^]^ Development of cell Lines expressing rs838133 was done using antibiotic selection. All constructs used in these studies were sequenced prior to experiments.

##### SiRNA

Transfection was performed with Lipofectamine RNAiMAX (Invitrogen) according to the manufacturer's instructions using 75 nm of ON‐TARGETplus human RPLP0 siRNA smart‐pool or ON‐TARGETplus Non‐targeting pool as a control for 48 h.

##### Ribonucleic Acid Extraction, First‐Strand Complimentary Deoxyribonucleic Acid Synthesis

Total RNA was extracted from tissue and cell lysates using the Qiagen RNeasy Kit according to the manufacturer's instructions. Complimentary DNA (cDNA) was reverse transcribed from total RNA using qScript cDNA SuperMix (Quanta Biosciences, Gaithersburg, MD, USA) according to the manufacturer's instructions.

##### Real‐Time Quantitative Polymerase Chain Reaction

Real‐time PCR was performed on Applied Biosystems, Foster City, CA, USA, using TaqMan Fam‐labeled expression probes with TaqMan Fast Advanced Master Mix or forward and reverse primers for each gene of interest with SYBR Select Master Mix and were normalized to the expression of glyceraldehyde 3‐phosphate dehydrogenase (GAPDH). Expression was measured using CT values normalized to that of GAPDH (ΔCT = CT [GAPDH] − CT [target]) and then expressed as 2^−ΔCT^. Primers sequences and expression probe IDs were provided in key resource table.

##### Allele‐Specific Expression

To explore whether rs838133‐allele‐specific expression contributes to FGF21 liver expression, the authors quantified both liver complimentary DNA (cDNA) and genomic DNA (gDNA) from 13 patients heterozygous for the variant using real‐time quantitative RT‐PCR.

##### Enzyme‐Linked Immunosorbent Assay

Concentrations of human FGF21, KLB, and FAP were quantified according to the manufacturer's instructions by using ELISA kits.

##### Liver Immunohistochemistry

4 *μ*m sections were mounted onto saline‐coated glass slides to ensure section adhesion through subsequent staining procedures. Slides were stained using the BOND RX Fully Automated Research Stainer (Leica Biosystems) using the Bond Polymer Refine Detection Method Algorithm as per manufacturer instructions (DS9800 Leica). The slides were incubated with a dilution of 1:200 rabbit monoclonal antibody against human anti‐FGF21 (FGF21 antibody [MA532652] size 100 UL, Invitrogen). Negative controls were carried out with rabbit serum diluted to the same concentration as the primary antibody. The extent of FGF21 immunostaining was assessed using ImageJ.

##### Western Blot

Whole cell extracts were fractionated by SDS‐PAGE and transferred to a polyvinylidene difluoride membrane using a transfer apparatus according to the manufacturer's protocol (Bio‐Rad). Following transfer, membranes were blocked with 5% non‐fat milk in TBST (50 mm Tris, pH 7.4, 150 mm NaCl, 0.05% Tween‐20) for 1 h at room temperature. Membranes were washed twice with TBST and incubated with primary antibodies diluted in 5% skim milk/TBST, overnight at 4 °C. Following incubation, membranes were washed thrice for 10 min with TBST and incubated with secondary antibodies, diluted in 5% skim milk/TBST, for 1 h in dark. Blots were washed with TBST thrice and developed with SuperSignal West Pico PLUS Chemiluminescent and SuperSignal West Femto Maximum Sensitivity Substrates (Life Technologies, Australia) according to the manufacturer's protocol. Membranes were scanned using ChemiDoc Touch Imaging system (Bio‐Rad).

##### Immunoprecipitation of Fibroblast growth factor 21 with Active Ribosomes

Huh7 cell lines that stably expressed *FGF21* isoforms representing the two alleles (A/G) of rs838133 (with or without SiRNA transfection) were treated with L‐leucine for 40 min then lysed and active ribosomes were isolated using the AHARIBO_Protein and AHARIBO_RNA kits (Immagina Biotechnology). Then, RNA and proteins associated to the active ribosomes were extracted directly from beads according to manufacturer's instructions.^[^ ^26^ ^]^ Following the isolation, RNA was reverse transcribed and amplified by RT‐PCR; proteins were resolved and subject to ELISA, as described above.

##### Measurement of Fibroblast Growth Factor 21 Protein Stability

To assess the impact of rs838133 polymorphisms on the rate of FGF21 degradation, Huh7 cells were transfected with pcDNA3.1 constructs containing the full‐length human FGF21, with genetic variation corresponding to A or G (of rs838133) using FuGENE HD (Promega), according to the manufacturer's instructions. On the next day, the cells were treated with vehicle (DMSO) or cycloheximide (CHX, 100 µg mL^−1^) over 4 h. Cell lysates were collected and FGF21 protein was measured by ELISA as per the manufacturer instructions. GraphPad Prism was used to fit the data to a one‐phase decay analysis.

##### Measurement of Fibroblast Growth Factor 21 mRNA Stability

To determine FGF21 mRNA half‐life, Huh7 cells stably expressing the A or G allele were treated with actinomycin D (Sigma) at a concentration of 10 µg mL^−1^. RNA samples were then isolated from the cells over a time course of 3 h and then subject to qPCR. One‐phase decay analysis on GraphPad Prism was performed to determine half‐life.

##### Polymerase Chain Reaction Microarrays

PCR microarrays were performed with The TaqMan Array, Human Transcription of tRNA, Fast 96‐well which contains 28 assays for transcription of tRNA‐associated genes and 4 assays to candidate endogenous control genes. Total RNA was extracted from the cells and measured by qPCR as per the manufacturer protocols. The authors utilized three different algorithms to predict changes in the RNA structure induced by rs838133, the RNAfold WebServer^[^ ^30^ ^]^ (compared the influence of the variant on the secondary structure of a gene), RNAsnp^[^ ^31^ ^]^ (provided *p*‐values reflecting the significance of local structure base‐pairing distance), and MutaRNA^[^ ^32^ ^]^ (provided a collection of measures and visualizations for an intuitive interpretation of the impact of variants on the RNA secondary structure).

##### Bioinformatics

Each codon was analyzed relative to the codon frequency within FGF21 CDS and the entire human genome. The codon usage frequencies of the human genome were obtained from Codon and Codon Pair Usage Tables (CoCoPUTs).^[^ ^63^, ^64^ ^]^ The RSCU was defined as the frequency of observed codons divided by the frequency expected under the hypothesis of uniform usage of all synonymous codons for a particular amino acid.^[^ ^65^ ^]^ RSCU values for FGF21 CDS were calculated by using R package “statanacoseq” and RSCU values for entire human genome were calculated via utilizing CoCoPUTs. RSCU values were divided into three categories: A) codons with RSCU values ≤0.6 (under‐represented), B) codons with RSCU values between 0.6 and 1.6 (unbiased‐represented), and C) codons with RSCU values ≥1.6 (over‐represented).^[^ ^66^, ^67^ ^]^ Glycine was encoded by four synonymous codons: GGG, GGA, GGC, and GGT.

Previously published and publicly available human microarrays from clinically defined normal, steatotic, and steatohepatitis patient livers were obtained from the ArrayExpress database (accession E‐MEXP‐3291) and analyzed for hepatic FGF21 expression correlation. Additionally, GEO datasets GSE32504 and GSE39036 were retrieved for the differential expression genes and pathways analysis. 148 liver samples of Caucasian origin were included in these studies in which GSE32504 provided the RNA expression data and GSE39036 provided the genotype information. R 3.6.2 was used to process the data obtained from these public datasets to generate the heatmap for hepatic FGF21 expression correlation and the volcano plot for differential gene expression. GSEA was performed in GSEA 4.0.3.^[^ ^68^ ^]^

##### Transfer Ribonucleic Acid Array

cDNA synthesis for tRNA PCR array: cDNA was synthesized by the rtStar tRNA‐optimized First‐Strand cDNA Synthesis Kit (Arraystar, Inc. Rockville, MD 20850, USA, Cat#: AS‐FS‐004) according to the manufacturer's instructions. This kit used reverse transcriptase at high temperature and without RNase H activity to ensure efficient reverse transcription of tRNA templates with strong secondary structures in addition to a demethylation step to efficiently remove m1A, m3C, and m1G modifications in the tRNAs which overcomes the well‐known problem that tRNAs undergo by far the greatest number of, and the most chemically diverse post‐transcriptional modifications. The kit used RNA spike‐in as an internal control for each reaction to monitor the cDNA synthesis efficiency and as a reference for qPCR data comparison.

##### Human Transfer Ribonucleic Acid Polymerase Chain Reaction Array

Human tRNA PCR arrays were performed with nrStar Human tRNA (Arraystar, Inc. Rockville, MD 20850, USA, Cat#: AS‐NR‐001H‐1) according to the manufacturer's instructions. The PCR array profiled 163 PCR‐distinguishable nuclear tRNA isodecoders and 22 PCR‐distinguishable mitochondrial tRNA species by optimized SYBR Green qPCR assays in a PCR panel. The panel covered all the anticodons compiled in the GtRNAdb and tRNAdb data base. 59 genes with Ct values equal to or more than 35 were considered as not detected and were not included in the analysis as recommended by the manufacturer data preprocessing quality control check procedure. The PCR array included a series of external and internal controls for correction and normalization of sample and qPCR variabilities. These controls included:

- Three reference controls for qPCR normalization which were stably expressed small ncRNA genes (U6, 5S rRNA and 18S rRNA).
- RNA Spike‐In (External Control): One external RNA spike‐in mix was added in the RNA sample prior to first strand cDNA synthesis. The RNA spike‐in control assay indicated the overall success and the efficiency of the reaction beginning from cDNA synthesis to the final qPCR.
- Positive PCR control: One artificial DNA and the PCR primer paired to indicate the qPCR amplification efficiency.
- gDNA Control: The control assay consisted of PCR primers for a non‐transcribed genomic region.

##### Patient Cohort

The study comprised 1209 Caucasian patients with biopsy‐proven MAFLD. The characteristics of the cohort and their clinical and laboratory assessment are provided in Table S2, Supporting Information. Ethics approval was obtained from the Human Research Ethics Committees of the Western Sydney Local Health District and the University of Sydney. All other sites had ethics approval from their respective ethics committees.

##### Genotyping

Genotyping for *FGF21* rs838133 was undertaken using the TaqMan SNP genotyping allelic discrimination method (Applied Biosystems, Foster City, CA, USA). All genotyping was blinded to clinical variables, as previously described.^[^ ^69^ ^]^

##### Liver Histopathology

Liver biopsies were scored by an expert liver pathologist in each participating center unaware of clinical or genetic data. Histological scoring was based on the system proposed by Kleiner et al.^[^ ^70^ ^]^ Steatosis was graded from zero to three, lobular inflammation from zero to three, and hepatocellular ballooning from zero to two. Fibrosis was staged from zero to four with four representing cirrhosis. The Activity Score (NAS) was calculated to quantify disease activity.^[^ ^70^ ^]^ The interobserver agreement between pathologists was studied previously and was excellent for steatosis (*κ* = 0.85) and good for fibrosis (*κ* = 0.78).^[^ ^71^ ^]^

##### Statistical Methods

Results were expressed as mean ± SEM (standard error of mean). The Student's *t*‐test or non‐parametric, Wilcoxon–Mann–Whitney *U*‐test, or Kruskal–Wallis tests were used to compare quantitative data, as appropriate. *χ* ^2^‐test and Fisher‐exact tests were used for comparison of frequency data and to evaluate the relationships between groups and the Cochran–Armitage test was used for trend. Multivariable logistic regression modeling was undertaken to test independent associations of the *FGF21* rs838133 variant with inflammation. All tests were two‐tailed and *p*‐values <0.05 were considered significant. Analysis was done by SPSS or GraphPad prism.

**Table 1:** Key resources table

**Reagent or resource**	**Source**	**Identifier**
**Antibodies**		
GAPDH, Mouse monoclonal antibody	Abcam	ab8245
FGF21, Rabbit monoclonal antibody	Life Technologies	MA5‐32652
Secondary antibody, Goat Anti‐Rabbit IgG H&L (HRP)	Abcam	ab6721
Secondary antibody, Goat Anti‐Mouse IgG H&L (HRP)	Abcam	ab6789
Phospho‐Akt (Ser473) (193H12) Rabbit mAb	Cell Signaling	4058
Akt Antibody Rabbit mAb	Cell Signaling	9272
Phospho‐p44/42 MAPK (Erk1/2) Rabbit mAb	Cell Signaling	4370
p44/42 MAPK (Erk1/2) Antibody	Cell Signaling	9102
**Bacterial and virus strains**		
NA		
**Chemicals, peptides, and recombinant proteins**		
Cycloheximide	Sigma	C7698
Actinomycin D	Sigma	A1410
**Critical commercial assays**		
TaqMan Array, Human Transcription of tRNA, Fast 96‐well	Thermo Fisher	4418841
Human KLB/Beta Klotho	Lifespan Bioscience	LS‐F39934
Human FAP ELISA Kit	Abcam	AB256404
Human FGF21 ELISA Kit	R&D Systems	DF2100
Automated IHC kit	Leica	DS9800
AHARIBO_Protein	Immagina Biotechnology	#AHA003_P
AHARIBO_RNA	Immagina Biotechnology	#AHA003_R
**Deposited data**		
NA		
**Experimental models: Organisms/strains**		
NA		
**Oligonucleotides**		
TaqMan SNP Genotyping Assay, human Assay ID: C‐8832415_10	Thermo Fisher	4351374
Assay ID: Hs00173927_m1/FGF21	Thermo Fisher	4331182
Assay ID: Hs02786624_g1/GAPDH	Thermo Fisher	4448489
Assay ID: Hs00221003_m1/FGF23	Thermo Fisher	4331182
Assay ID: Hs01653088_g1 Gene Symbol: RPLP1 Dye	Thermo Fisher	4448892
Assay ID: Hs01115128_gH Gene Symbol: RPLP2	Thermo Fisher	4448892
Assay ID: Hs99999902_m1 Gene Symbol: RPLP0	Thermo Fisher	4331182
TaqMan Array, Human Transcription of tRNA, Fast 96‐well	Thermo Fisher	4418841
Human GAPDH primer	NA	Forward: AAGGTGAAGGTCGGAGTCAAG Reverse: GGGGTCATTGATGGCAACAATA
Human TNF‐A primer	NA	Forward: TCTCTAATCAGCCCT Reverse: TACAACATGGGCTAC
Human CCL2 primer	NA	Forward: TGC CGC CCT TCT GTG CCT G ACA GCA GGT GAC TGG GGC AT
Human CXCL10 primer	NA	Forward: TGCAAGTCTATCCTGTCCGC Reverse: TCTTTGGCTCACCGCTTTCA
Human EGR1 primer	NA	Forward: AGCAGCACCTTCAACCCTCAGG Reverse: GAGTGGTTTGGCTGGGGTAACT
Human c‐fos primer	NA	Forward: CTGGCGTTGTGAAGACCAT Reverse: TCCCTTCGGATTCTCCTTTT
**Software and Algorithms**		
ImageJ	BD	https://imagej.nih.gov/ij/
Graphpad Prism 7	Graphpad	Graphpad
R 3.6.2.	R	https://www.r‐project.org
GSEA 4.0.3.	GSEA	https://www.gsea‐msigdb.org/gsea/index.jsp
**Other**		
RNeasy Kit	Qiagen	74106
qScript cDNA SuperMix	Quanta Biosciences	95048
TaqMan Fast Advanced Master Mix	Thermo Fisher	4444963
FuGENE HD Transfection Reagent	Promega	Catalog number: E2311
ON‐TARGETplus Human RPLP0 siRNA smartpool	Dharmacon	L‐010864‐00‐0005
ON‐TARGETplus Non‐targeting Pool	Dharmacon	D‐001810‐10‐05
Lipofectamine RNAiMAX Transfection Reagent	Invitrogen	13778150
SuperSignal West Pico PLUS Chemiluminescent and SuperSignal West Femto Maximum Sensitivity Substrates	Life Technologies	34580, 34096

## Conflict of Interest

The authors declare no conflict of interest.

## Supporting information

Supporting Information

## Data Availability

All data are available within the main text and Supporting Information. This study did not generate any unique datasets or code.
