# Supplementary material for: Mistranslation Drives Alterations in Protein Levels and the Effects of a Synonymous Variant at the Fibroblast Growth Factor 21 Locus
Source: Adv Sci (Weinh). 2021 May 1;8(11):2004168. doi: 10.1002/advs.202004168 (PMC8188187; doi:10.1002/advs.202004168)
Supplement: Supplementary file 1 — Supporting Information [file ADVS-8-2004168-s001.pdf]

© 2021 Wiley-VCH GmbH

ADVANCED SCIENCE
Open Access

Supporting Information

for *Adv. Sci.,* DOI: 10.1002/advs.202004168

Mistranslation drives alterations in protein levels and the effects of a synonymous variant at the FGF21 locus

Ali Bayoumi, Asmaa Elsayed, Shuanglin Han, Salvatore Petta, Leon A. Adams, Rocio Aller, Anis Khan, Carmelo García-Monzón, María Teresa Arias-Loste, Luca Miele, Olivier Latchoumanin, Shafi Alenizi, Rocio Gallego-Durán, Janett Fischer, Thomas Berg, Antonio Craxì, Mayada Metwally, LiangQiao, Christopher Liddle, Hannele Yki-Järvinen, Elisabetta Bugianesi , Manuel Romero-Gomez, Jacob George and Mohammed Eslam**

Mistranslation drives alterations in protein levels and the effects of a synonymous variant at the FGF21 locus

Ali Bayoumi, Asmaa Elsayed, Shuanglin Han, Salvatore Petta, Leon A. Adams, Rocio Aller, Anis Khan, Carmelo García-Monzón, María Teresa Arias-Loste, Luca Miele, Olivier Latchoumanin, Shafi Alenizi, Rocio Gallego-Durán, Janett Fischer, Thomas Berg, Antonio Craxì, Mayada Metwally, LiangQiao, Christopher Liddle, Hannele Yki-Järvinen, Elisabetta Bugianesi, Manuel Romero-Gomez, Jacob George and Mohammed Eslam.

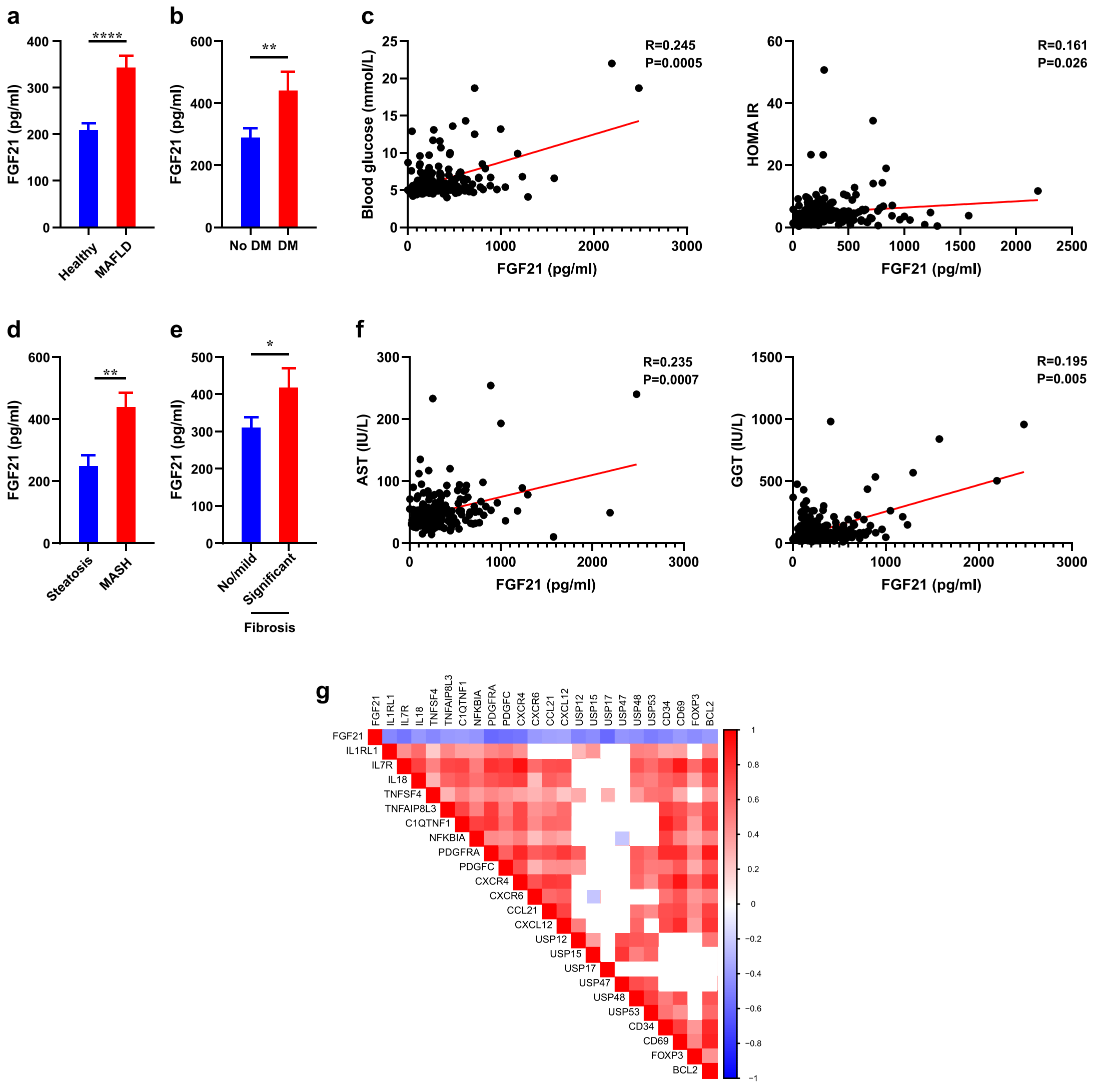

Supplementary Figure 1: Serum FGF21 levels. (**a**) FGF21 serum levels were significantly increased in patients with MAFLD compared to healthy controls. (**b**) FGF21 serum levels were higher in diabetic compared to non-diabetic patients. (**c**) FGF21 serum levels positively correlated with glycaemic parameters. A significant positive correlation between FGF21 serum levels and blood glucose and HOMA-IR (**d**) FGF21 serum levels were significantly increased in patients with metabolic steatohepatitis compared to those with simple steatosis. (**e**) FGF21 serum levels were significantly increased in patients with fibrosis (F2-F4) compared to those with no or mild fibrosis (F0-F1). (**f**) FGF21 serum levels and their correlation with liver enzymes. There was a significant positive association between FGF21 serum levels and AST and GGT. (**g**) To determine if hepatic FGF21 influences inflammation in a clinical context, Pearson correlations were performed between hepatic FGF21 mRNA and all measured chemokines/inflammatory cytokines and receptors in publicly available microarray data from healthy liver samples. A subset of chemokines of this correlation is displayed. Statistical differences between groups were assessed by the t-test or the Spearman correlation coefficient. The data presented are mean ± sem or expressed as R and P, respectively. (* $p < 0.05$, ** $p < 0.01$).

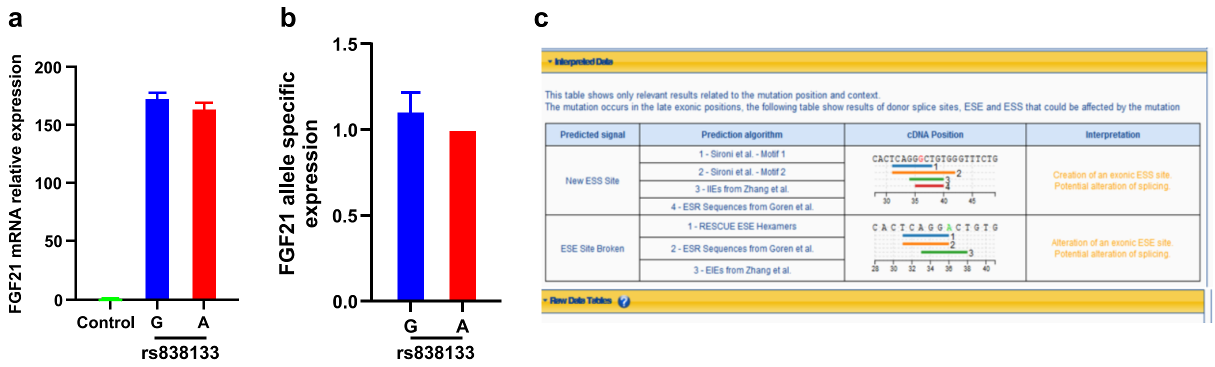

Supplementary Figure 2: The rs838133 does not affect mRNA levels. (**a**) Huh7 cells stably expressing the A or G alleles of rs838133 in FGF21 were analysed by qRT-PCR normalised to GAPDH in the ribosomal fraction. (**b**) FGF21 rs838133 has no effect on hepatic allele specific expression (ASE). Allele-specific ratio quantified by real-time PCR in subjects heterozygous for rs838133. No difference in the expression of the minor (A) (n = 13) compared with the major allele (G) (n = 13, p = 0.3) was observed. Statistical differences between groups was assessed by the unpaired t test; the data are presented as mean ± sem. (**c**) The rs838133 SNP does not affect mRNA splicing using an exonic splicing enhancer finder.

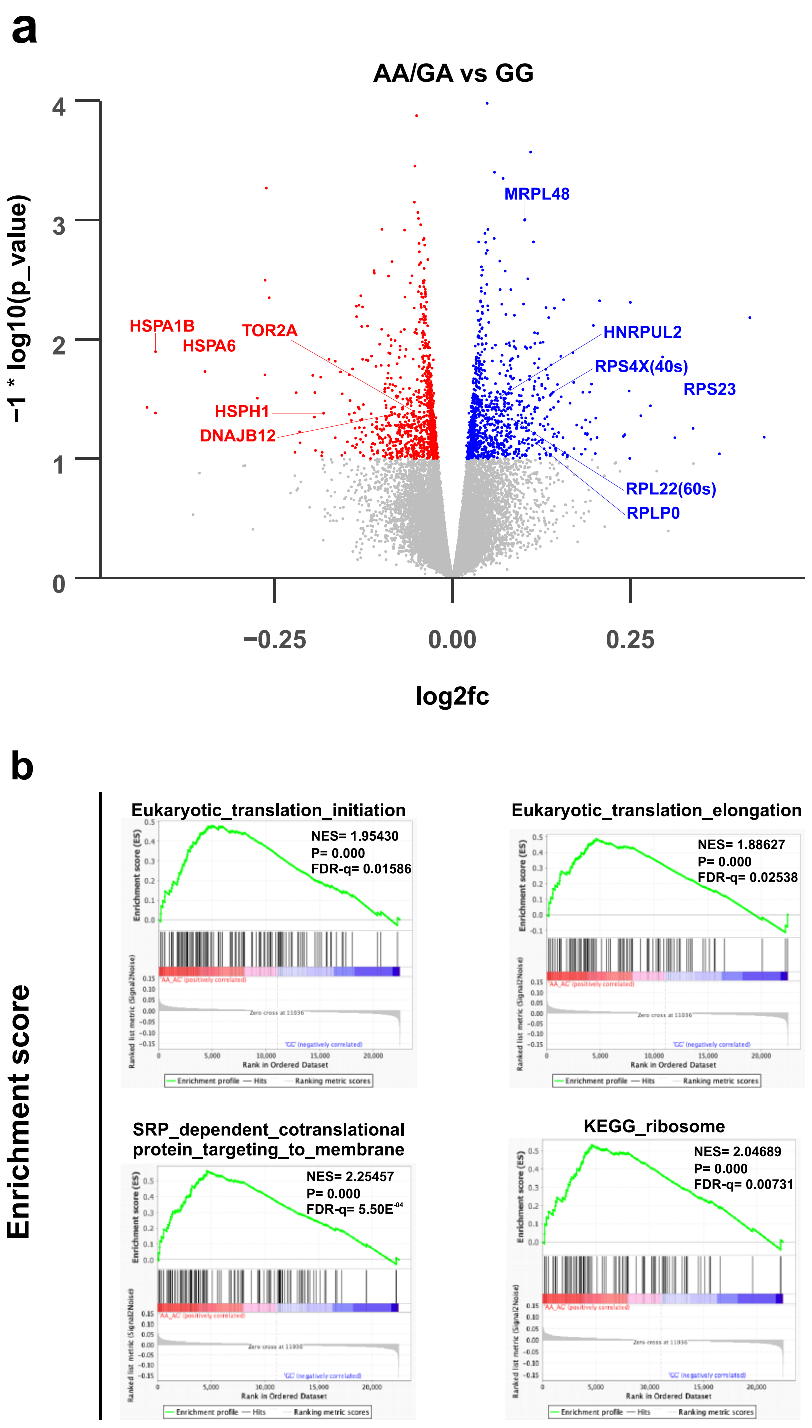

Supplementary Figure 3: **a)** Volcano plot of differential translation gene expression between (A) allele and two copies of the (G) allele (**b**) and Gene Set Enrichment Analysis (GSEA) analysis for the NCBI Gene Expression Omnibus (GEO) database (GEO: GSE32504 and GSE39036) that contains transcriptomic and genotype profiles, respectively, of 149 liver samples of Caucasian origin subjects shows overrepresentation of genes encoding translation constituents in the (A) allele versus those with two copies of the (G) allele including translation initiation and translational regulation, co-translation and ribosomal proteins.

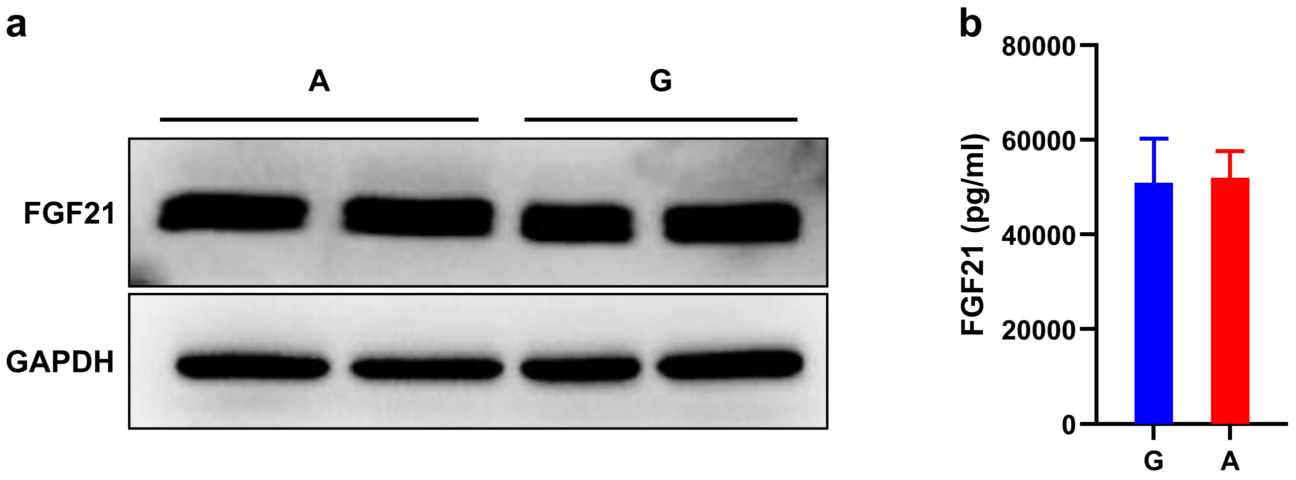

Supplementary Figure 4: (**a**) Cells were transiently transfected with the G or A allele during the cycloheximide chase assay; the amount of total FGF21 by Western blot analysis is displayed, (**b**) or the amount of FGF21 protein by ELISA. Statistical differences between groups was assessed by the unpaired t test for three independent replicates; the data are presented as mean ± sem.

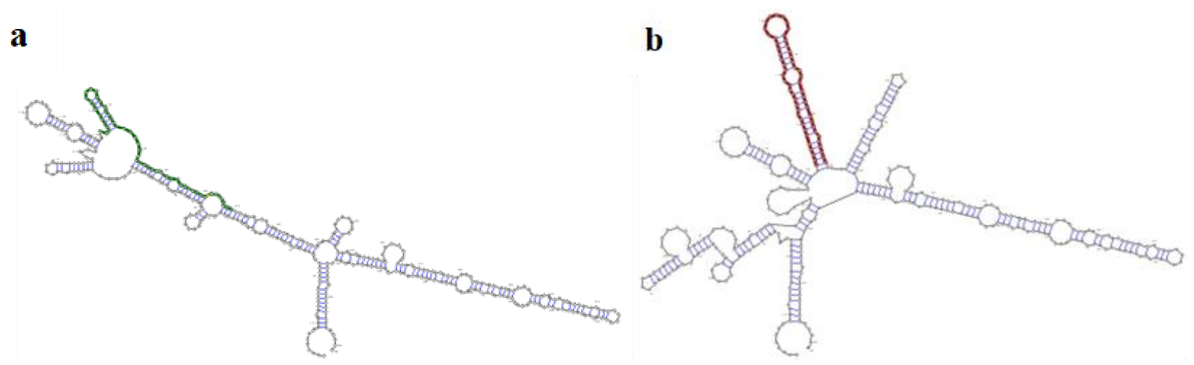

Supplementary Figure 5: The genotype of rs838133 affects the mRNA secondary structure and expression of FGF21. The mRNA secondary structures of full-length FGF21 with the (**a**) rs838133 (A) allele or (**b**) the rs838133 (G) allele are significantly different (p=0.02) as predicted using the RNASNP.

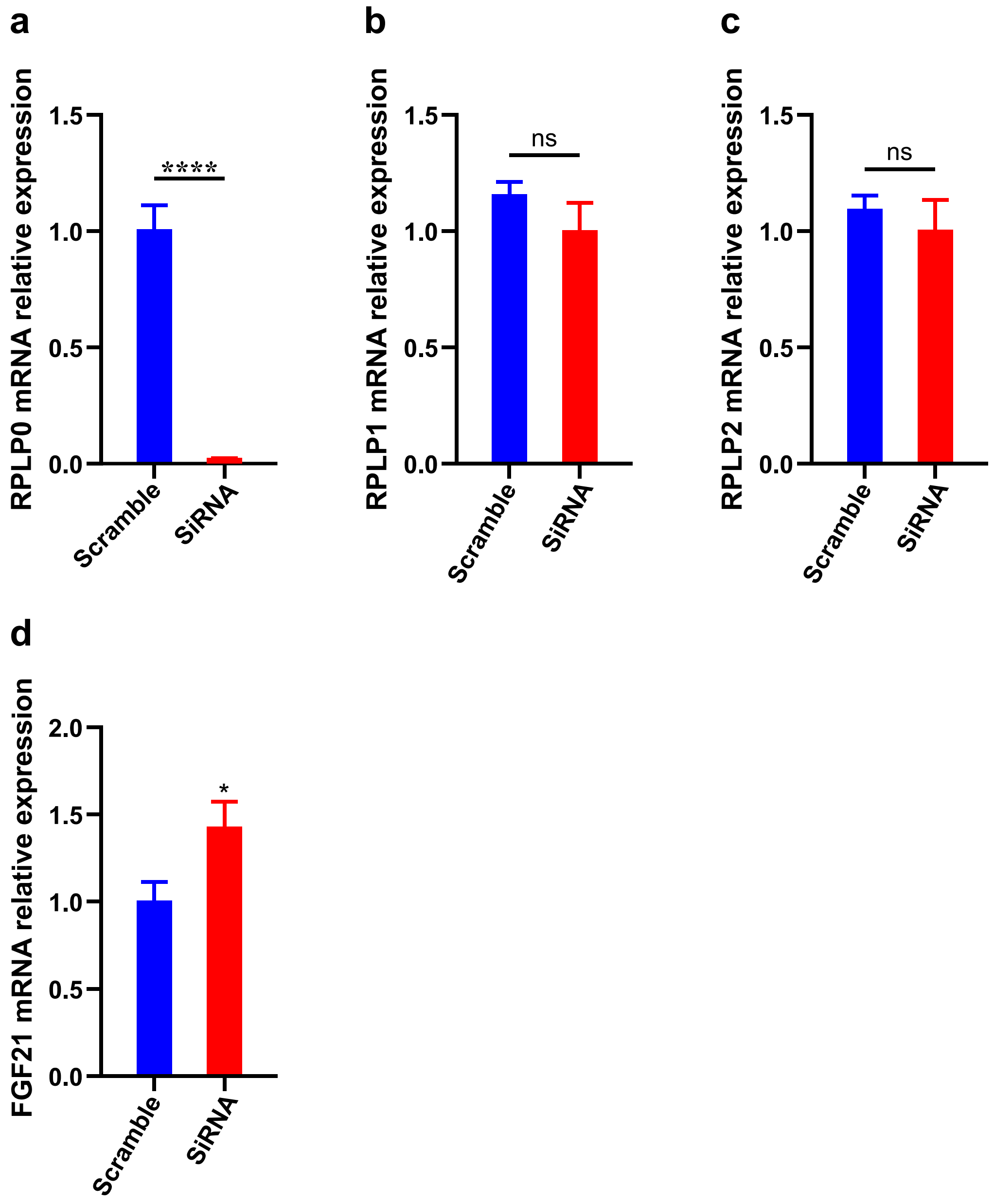

Supplementary Figure 6: RPLP0 siRNA. mRNA expression of RPLP0 (**a**), RPLP1 (**b**) and RPLP2 (**c**) analysed by qRT-PCR normalised to GAPDH in cells transfected for 48 h with RPLP0-specific or control siRNA probes. RPLP0 inhibition by siRNA led to modest induction of FGF21 mRNA expression (**d**). Statistical differences between groups was assessed by the unpaired t test for three independent replicates; the data are presented as mean ± sem. (ns, non-significant, *$P < 0.05$, ** $p < 0.01$, *** $p < 0.001$).

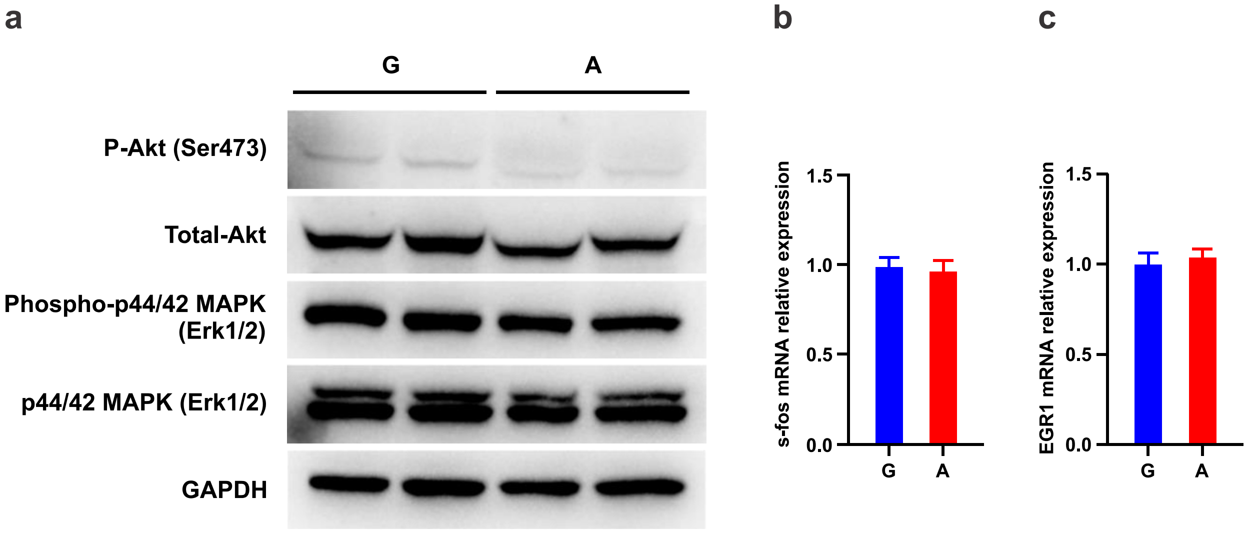

Supplementary Figure 7: The FGF21 downstream signaling cascade in Huh7 cells stably expressing the A or G alleles of rs838133 analysed by (**a**) Western blot for the phosphorylation of ERK and AKT and RT-PCR for the mRNA expression of (**b**) cFos and (**c**) Egr1

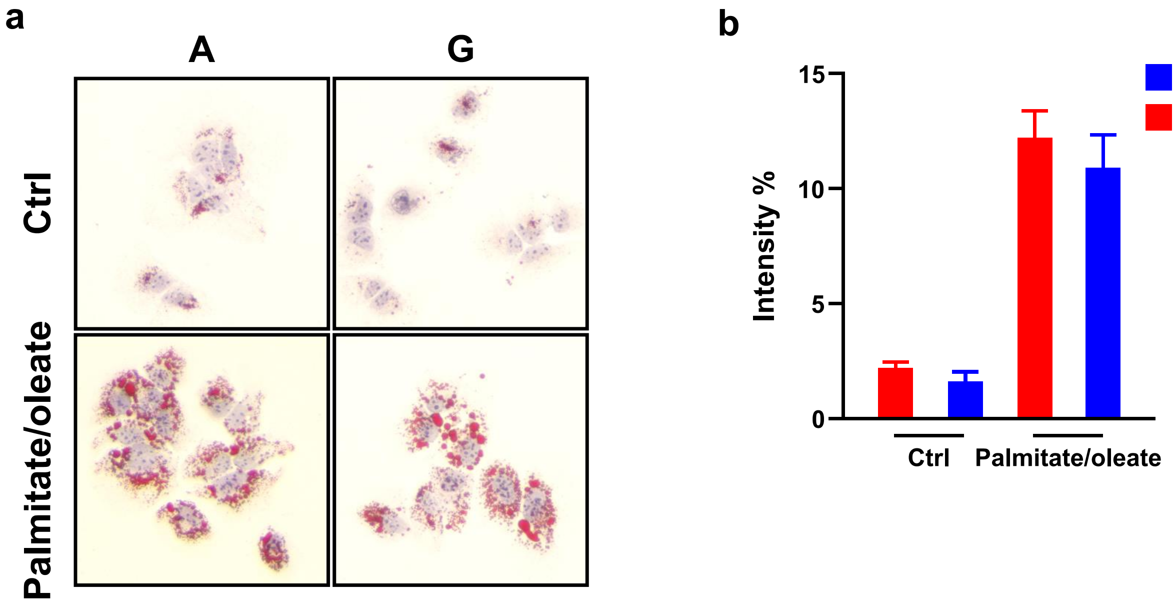

Supplementary Figure 8: The impact of rs838133 on intracellular hepatocyte lipid accumulation. (**a**) Lipid droplet content visualized by ORO-staining in Huh7 cells stably expressing the A or G allele incubated with palmitic acid and oleate (200 and 400 uM, respectively) for 24 hours. (**b**) ORO-stained area quantified by imageJ. Values of three independent replicates are represented by vertical bars and are mean ± sem; *$P < 0.05$ using the student t-test.

Supplementary Table 1: Relative synonymous codon usage (RSCU) values of glycine coding codons in the human genome and FGF21 cDNA.

Variant	Codon	Human Genome	FGF21 CDS
WT	GGA	1.091	1.636
c.36A>G	GGG	0.969	1.273
c.36A>T	GGT	0.687	0.364

Supplementary Table 2: Demographic, anthropometric and clinical characteristics of the patient cohort with MAFLD

	**MAFLD cohort (n = 1209)**
**Age (yrs)**	48 (38-58)
**Male (%)**	640 (52.9)
**BMI (Kg/m^2)**	30.8 (27.6-36.6)
**ALT (IU/L)**	53 (32-88)
**AST (IU/L)**	35 (24-51)
**GGT (IU/L)**	54 (31-105)
**Platelet ($x10^9$/L)**	241 (198-287)
**Diabetics (%)**	297 (24.6)
**Cholesterol (mmol/L)**	4.93 (4.22-5.7)
**Triglycerides (mmol/L)**	1.48 (1.05-2.19)
**HDL-C (mmol/L)**	1.2 (1-1.5)
**LDL-C (mmol/L)**	2.9 (2.3-3.6)
**Blood glucose (mmol/L)**	5.4 (4.9-6.38)
**HOMA-IR**	3.2 (2.007-5.18)

Data are presented as median and range. HOMA-IR, Homeostatic Model Assessment of Insulin Resistance. BMI; body mass index.

Supplementary Table 3: Distribution of *FGF21* rs838133 genotype and Hardy-Weinberg equilibrium.

***FGF21* rs838133**	**MAFLD (n =1209)**
AA	235
AG	556
GG	418
A allele	43%
G allele	57%

Table 3. 1. Distribution of FGF21 rs838133 genotype and Hardy-Weinberg equilibrium.

$p = 0.1$. P values were calculated by chi square test, $p > 0.05$ indicates no deviation from Hardy-Weinberg equilibrium.
